# Effect of Posture Training with Weighted Kypho-Orthosis (WKO) on Improving Balance in Women with Osteoporosis

**DOI:** 10.1155/2014/427903

**Published:** 2014-03-06

**Authors:** Seyed Ahmad Raeissadat, Leyla Sedighipour, Safura Pournajaf, Reza Vahab Kashani, Shahram Sadeghi

**Affiliations:** ^1^Department of Physical Medicine & Rehabilitation, Clinical Research Development Center of Shahid Modares Hospital, Shahid Beheshti University of Medical Sciences, Tehran, Iran; ^2^Department of Orthotics and Prosthetics, University of Social Welfare and Rehabilitation Science, Kodakyar street, Daneshjo Boulevard, Evin, Tehran 1985713834, Iran

## Abstract

*Objectives.* To determine the effect of weighted kypho-orthosis (WKO) on improving balance in women with osteoporosis. In this nonrandomized controlled clinical trial, 31 patients with osteoporosis were included. The patients were assigned to two groups: (1) control group who received 4-week home-based daily exercise program including weight bearing, back strengthening, and balance exercises and (2) intervention group (WKO) who performed aforementioned exercises and wore WKO for one hour twice a day. Patients were assessed using clinical balance tests (timed up and go test, functional reach test, and unilateral balance test) before and 4 weeks after start of treatment. *Results.* Functional reach and timed up and go test were improved significantly in both groups compared to baseline. The improvement in intervention group was more significant in comparison to control group (*P* < 0.05). *Discussion.* Posture training with WKO together with exercise program improved two clinical balance tests in women with osteoporosis. *Conclusion.* Posture training support (PTS) applied as WKO together with back extension exercises can be prescribed as an intervention in elderly women in order to reduce the risk of falling.

## 1. Introduction

Osteoporosis is the most common metabolic bone disease in the world [1–3]. The most common clinical consequences are back pain, hyperkyphosis, limitations of physical functioning and activities of daily life, and impaired health-related quality of life in osteoporotic patients [4–6]. It is characterized by low bone mass and microarchitectural deterioration of bone tissue, leading to increased bone fragility and a consequent increase in fracture risk [1, 2, 7]. Vertebral fractures are the main clinical consequences of spinal osteoporosis and constitute a major public health burden. The incidence of spine fracture is estimated to be 20% in postmenopausal women [4]. The weakness of paraspinal extensor muscles noticed in osteoporosis accompanied by repetitive vertebral microfracture results in progressive change in alignment of the spine, mainly hyperkyphosis, in osteoporotic patients [2, 8–10]. The postural changes and vertebral fractures impose additional physical stress on the mechanically less robust vertebral bodies leading to acute and chronic back pain [5]. Furthermore, osteoporosis is associated with deficits of gait and balance, all together resulting in an increased risk of falls and a subsequent aggravation of fracture risk [7]. Falls among the elderly, especially for those with osteoporosis, are associated with high morbidity and mortality and can involve high-cost medical intervention [11]. In fact, falls are responsible for 90% of the growing increase in hip fractures [11] and are the sixth cause of death among patients aged over sixty.

Fall prevention is a necessity to reduce health costs in the senile population [11]. There are studies indicating that almost one-third of falls followed by detrimental consequences are preventable [12]. Apart from environmental modifications, improvements in internal factors such as static and dynamic posture and balance training are among most precious preventive strategies. Beside pharmaceutical therapy, physical rehabilitative measures play a key role in prevention of falls. Muscle reeducations and resistance exercises for strengthening and reduction of kyphosis are key elements for reducing the risk of falls and subsequent fractures [2, 13–15].

The flexed posturing in kyphotic patients caused by osteoporosis places their center of gravity closer to its limits of stability and increases the risk of falling [16, 17]. Therefore, measures that can improve axial stability can decrease disequilibrium and increase the risk of falls.

Previous studies have shown that wearing a spinal orthosis such as osteomed, spinomed, and WKO has a role in reduction of pain as well as improvements of posture and back extensor strength. WKO is among the most known orthosis in osteoporotic patients. In 2005, Sinaki et al. conducted a study to evaluate the effects of WKO on gait disturbances and risk of falls in patients with osteoporosis [18]. In the mentioned study, she indicated the positive effects of WKO together with spinal proprioceptive extension exercise on balance, gait, and risk of falls assessed by computerized dynamic posturography.

Most of the mentioned studies mainly focused on para clinic and laboratory based computerized tests to evaluate the efficacy of WKO on risk of falling. To date, there is no study having evaluated the effects of WKO on balance and gait stability via functional clinical tests in patients with osteoporosis. Therefore the purpose of the present study was to assess the effects of wearing WKO together with spinal exercises on balance via performing functional clinical tests in women with osteoporosis.

## 2. Methods

This study was conducted between 2010 and 2011 in Shahid Modarres Hospital, Tehran, Iran. The protocol of this experiment was approved by Shahid Beheshti University of Medical Sciences, Human Ethics Research Committee. Participants were patients voluntarily referred to physical medicine and rehabilitation clinic. All patients were screened by experienced clinicians skilled in the evaluation and the management of osteoporotic patients.

Inclusion criteria were as follows: women with osteoporosis documented by low bone mineral densitometry at spine (T-score of <−2.5 aged between 55 and 75 years, thoracic kyphosis angle between 35 and 55 degree (Cobb's angle of thoracic kyphosis was calculated from lateral radiograph of spine)).

The patients with the following criteria were excluded:

history of secondary osteoporosis due to metabolic diseases (such as hyperthyroidism) evaluated by clinical laboratory tests, the presence of neuromuscular disorders, history of vertebral fracture in last 6 months, and history of spinal or lower limb surgical interventions including arthroplasty, vertebroplasty, and discectomy.

Patients who met the above-mentioned criteria were assigned to two groups (intervention and control groups).

### 2.1. Recruitment

This study was designed a non randomized clinical trial. Patients were recruited by convenient sampling. Patients were screened initially by physiatrists to determine whether they met the inclusion criteria. Those who met inclusion criteria and were willing to participate in the study were scheduled to wear the orthosis as intervention group.

At the same clinic, like orthosis group, matched controls of comparable age and weight and height were enrolled in the study as control group.

Patients in both groups, that is, orthosis and control groups, received medications including 1000 mg calcium citrate or carbonate and 400 IU vitamin E daily and sodium alendronate 70 mg weekly.

Patients in both groups were also instructed to have 30 minutes of daily walking.

Patients were followed via telephone calls to home to make sure that they were sticking to the exercises correctly as prescribed for them.

### 2.2. Control Group

No orthosis was prescribed for this group, but patients were taught to perform back extensor strengthening exercises at home (including 10 contractions of back extensors without increasing the low back lordosis) based on professor Sinaki program in Mayo clinic [19] as follows.

1. In supine position, the patient flexes the knees and then abducts and adducts the shoulders, while the elbows are extended and upper limbs are in contact with the floor.
2. In the supine position, the patient flexes the knees and puts hands over the head. By contracting abdominal muscles the patient tries to draw the lowback to the floor and keep this position for 10 seconds.
3. In the prone position, the patient puts a pillow under the lower abdomen and gently raises the head and shoulders from the floor as much as possible and keeps this position for 10 seconds.
4. The patient sits on a chair and puts the hands behind the head. Then he/she moves the elbows behind the head while inhaling. The patient will do the opposite while exhaling.
5. Sitting on a chair, the patient flexes the elbows while keeping the arms near to the trunk and then moves the arms back to reduce thoracic kyphosis.

All of the exercise programs were taught by an experienced physical therapist. In addition, an illustrated paper describing each exercise was presented to each participant. Besides, each patient was given a paper with a table and was asked to specify every day if he/she would do the exercises and bring it on followup visits. The patients were called by the educating physical therapist every week to assess exercise performance and associated problems. After 4 weeks, patients were called to return to hospital for reevaluations.

### 2.3. Intervention Group (Orthosis Group)

Patients in this group were guided to perform exercises the same as control group.

WKO was also administered for the patients in this group.

### 2.4. Weighted Kypho-Orthosis (WKO)

**Figure 1:**
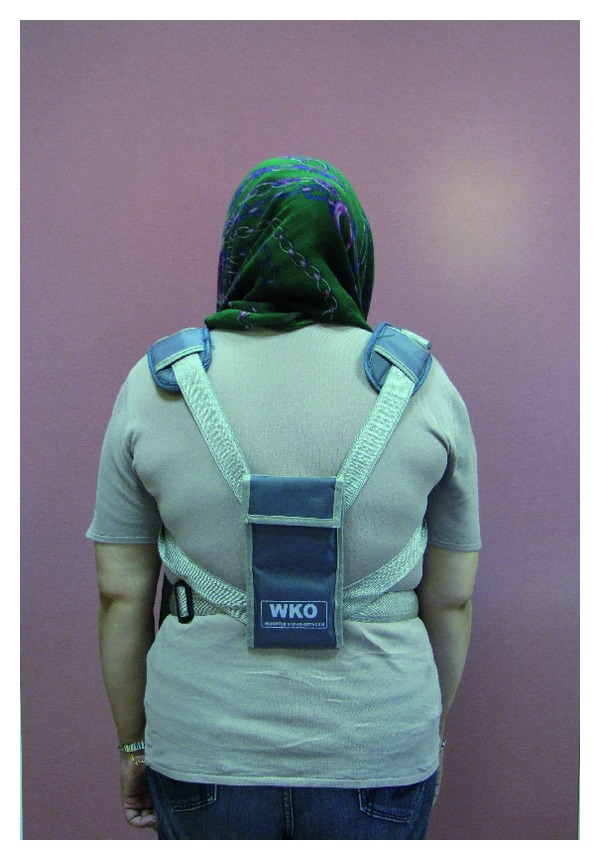
Patient wearing WKO (back view).

**Figure 2:**
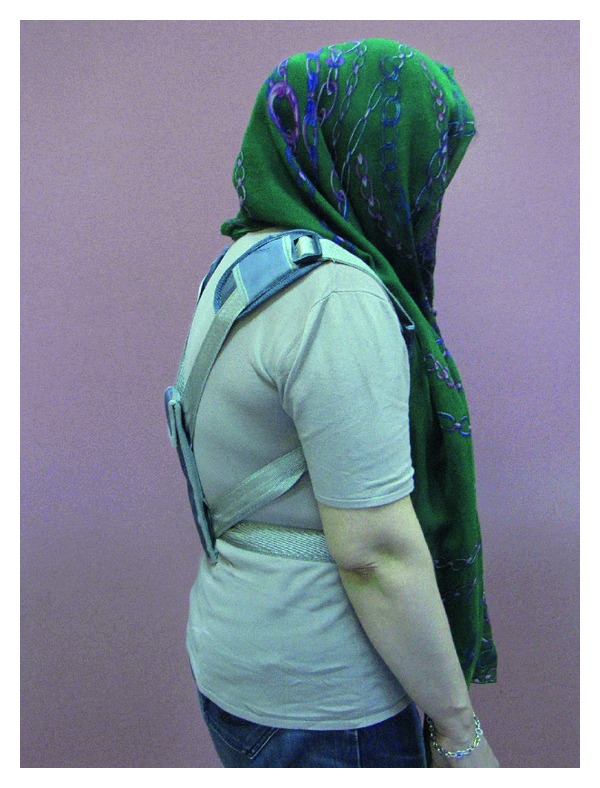
Patient wearing WKO (side view).

Weighted kypho-orthosis is an especially designed orthosis (made by TechnoTan company) with a harness and a 2-pound pouch, which centers its weight on the posterior of the spine at T10 to L4 (Figures 1 and 2).

The patients were guided to place the WKO over the thoracic spine and adjust the straps such that the bottom of the pouch is located at the waistline.

Patients were instructed to wear the device when ambulating for one hour a day (30 minutes at the morning and the evening) for 4 weeks. During those 4 weeks, patients were followed by telephone calls to assure that they were using the orthosis correctly.

An experienced orthotist instructed the patient regarding the proper usage of orthosis.

## 3. Measures

Dual X-ray absorptiometry (DXA) was used for measuring bone mineral density of spine or hip. All patients were referred to one imaging center for bone mineral density.

Cobb's angle of kyphosis is calculated from perpendicular lines drawn on a standard thoracic spine radiograph: a line extends through the superior endplate of the vertebral body, marking the beginning of the thoracic curve, and the inferior endplate of the vertebral body, marking the end of the thoracic curve.

Osteoporotic fractures were ruled out via evaluating X-rays by an experienced radiologist.

Functional tests which were performed at study entry were repeated 4 weeks after initiating therapy in both groups.

The same therapist performed the functional tests before and after therapy.

### 3.1. Functional Reach Test (FR)

Functional reach was tested by placing a yardstick or tape measure on the wall, parallel to the floor, at the height of the acromion of the subject's dominant arm. The subject was asked to stand with the feet in a comfortable distance apart, make a fist, and forward flex the dominant arm to approximately 90 degrees. The subject was asked to reach forward as far as possible without taking a step or touching the wall. The distance between the start and end point was then measured using the head of the metacarpal of the third finger as the reference point [20].

### 3.2. Timed Up and Go Test (TUG)

We asked the patients to wear their usual footwear and be seated in a chair with their back to the chair and their arms resting on the arm rests, and then we asked the patient to stand up from that chair and walk a distance of 10 ft. (3 m), then turn around, walk back to the chair, and sit down again.

Timing began when the person started to rise from the chair and ended when he or she returned to the chair and sits down.

The person was given 1 practice trial and then 3 actual trials. The durations from the three actual trials were averaged.

### 3.3. Unilateral Stance Test (US)

This test was performed in the standing position without foot wear with the patients' arms set by their sides and with open eyes. Timing was started when the subjects raised the appropriate foot off the ground. Timing was stopped if subjects displaced the foot they were standing on, touched the suspended foot to support the weight bearing limb, or reached the maximum balance time of 30 seconds [21, 22]. Third trial was performed if the maximum balance time was not reached in either of the first two trials. The longest trial with eyes open was used for data analysis. The length of each trial was ten seconds.

## 4. Data Analysis

SPSS-18 was used for data analysis. According to kolmogorov-smirnov normality tests, all variables including demographic data and the time of functional tests were normally distributed and therefore parametric tests including independent *t*-test and paired *t*-test were conducted to determine if there was a statistically significant change in demographic characteristics between the two groups and improvement in functional tests after therapy.

The assessors including the statistician and the physical therapist who observed the patients during functional tests and recorded the results were blinded to which group the patients belonged to.

## 5. Ethics

From the ethical point of view, the patients were enrolled after providing informed consent as approved by the institutional review board of Shahid Beheshti Medical University study. The process of treatment was explained completely to the patients and once the physician assured that the patient completely understood the study protocol and became aware of his or her rights during the study, the written consent form was signed or fingerprinted by the patient. The process of treatment had no harm for their health, and they had authority to stop the process of treatment liberally. The control group had also received routine medication and exercise therapy as treatment. Participants of this study were not charged for being involved in the study or using orthosis.

## 6. Results

The patients' characteristics at the start of study are displayed in Table 1. There were no between-group differences at the baseline in demographic characteristics and functional balance tests (Table 1).

**Figure 3:**
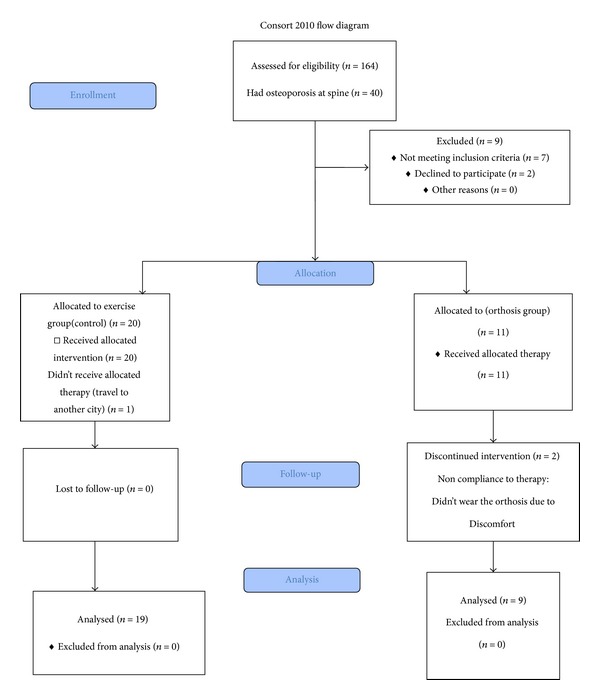
CONSORT 2010 Flow Diagram.

At the beginning, 164 osteoporotic patients (hip and/or spine) were assessed for eligibility to enter the study. From 40 patients with osteoporosis at spine, 31 patients met the inclusion criteria and accepted to participate in the study, and 28 people stayed at the program during the followup (two persons from orthosis and one person from control group were dropped out of the study due to incomplete exercises and not wearing the orthosis completely). The results of 28 patients (19 patients in control and 9 patients in intervention group) who remained in the study were analyzed. (CONSORT flow chart, Figure 3).

The mean of T score in control group was −2.74 ± 0.2 and the mean T-score of the intervention group was −2.99 ± 0.2 and there was no difference between two groups (*P* > 0.05).

Kyphosis angle's mean of the control group was 48.6 ± 5.5 degree and for the intervention group was 45.8 ± 5.7 degree and there was no difference between the two groups (*P* > 0.05).

### 6.1. Timed Up and Go Test (TUG)

As it can be read from Table 2, there was no statistically significant difference in this test scores between the two groups at the start of study; but at the end of study, patients in both intervention and control groups improved significantly regarding the scores of this test. This improvement was significantly more noticeable in intervention group compared to control group (*P* = 0.005).

### 6.2. Functional Reach Test (FR)

The scores of this test was not statistically different between the two groups at baseline, the scores of this test improved in both intervention and control groups significantly at the end of study.

However, patients in intervention group improved more significantly compared to control group (*P* = 0.005) (Table 2).

### 6.3. Unilateral Stance Test (US)

There was not any between-group differences regarding the scores of this test at baseline; but at the end of the study, the scores of this test improved significantly only in control group (Table 2).

## 7. Discussion

According to the results of the present study, improvement in balance was noticed in patients with osteoporosis after wearing WKO for 4 weeks according to TUG and FR tests.

Functional reach is a valuable tool for identifying those elderly with a high risk of falling. This test is designed to evaluate anteroposterior stability by measuring the maximum distance that a person can reach forward while standing over a fixed base of support. It assesses the ability to remain stable in this plane of movement [23].

TUG test included many components of other functional tests such as sit-to-stand, gait, and turning which requires many aspects of postural control; therefore the predictive value of this test for falling is high [24, 25]. This test was introduced as a basic test for functional mobility evaluation in 1991 [26, 27].

TUG is the simplest and probably the most reliable clinical test in evaluating balance [28]. Additionally, previous studies have found that TUG can discriminate between faller and nonfallers; therefore this test can be considered as a screen measure to predict falling in the elderly [25, 29]. This is mainly due to the fact that TUG is the only measure that includes a gait component which is functionally important because many falls occur during ambulation [25]. It is a valid method for screening of both level of functional mobility and risk of falling in community-dwelling elderly people [30].

TUG and FR both have high specificities (100% and 92%, resp.) [31].

Considering good predictive value of FR and TUG tests for falls, the improvement of these test scores after wearing WKO in our study can be suggestive of decreased risk of fall.

There are few studies evaluating the beneficial impacts of spinal orthosis on gait balance and reducing the risk of fall-in old aged population [9, 18, 32–34].

In 1996, Kaplan and Sinaki [9] evaluated effect of back supports on back strength in patients with osteoporosis in a pilot study. Patients were assigned to three groups (1) postural exercise only, (2) postural exercise and a conventional thoracolumbar support, or (3) postural exercise and a posture training support (PTS) (a WKO). Compliance with the use of the WKO was better than the one with the thoracolumbar support. Back extensor strength increased in patients who complied with the WKO and postural exercise program.

In 2005, Sinaki et al. [18] conducted a study to determine the outcome of intervention with a spinal weighted kypho-orthosis (WKO) and a spinal proprioceptive extension exercise dynamic (SPEED) program on the risk of fall-in osteoporotic patients. After 4 weeks of intervention, balance, gait, and risk of falls assessed by computerized dynamic posturography improved significantly with the 4-week SPEED program.

The role of exercise in the treatment of osteoporosis is to improve axial stability through improvement of muscle strength. Therapeutic exercise should address osteoporosis-related deformities of axial posture, which can increase risk of fall and fracture [35].

In our study, the beneficial effect of exercises was shown as patients in exercise group (control group) improved significantly based on FR, TUG, and US tests compared to baseline, even though this improvement was less than WKO regarding FR and TUG tests.

WKO promotes improvement in posture and increases back extensor strength by two mechanisms: first, the device produces a posterior force below the inferior angle of scapula and reduces anterior compressive forces exerted on the kyphotic spine [10]. Second, application of the WKO increases a patient's perception of spinal joint position, which plays an important role in static and dynamic posture. It creates a preprioceptive input and enhances the patient's ability to sense the position of the spine, which has an important role in strengthening the back extensor muscles and leads to improvement in static and dynamic postural stability [18]. WKO also promotes muscle reeducation and decreases painful contractions of the erector spinae muscles in kyphosis due to decrease in the load over the anterior aspect of the spinal column and vertebral bodies through use of a WKO [36].

Besides WKO, there are some studies investigating other types of spinal orthoses in osteoporosis. In 2008, Vogt et al. [32] investigated the efficacy of a flexible spinal orthosis without any stabilizing components in posture improvement. Forty women were randomized to three treatment groups: (1) osteomed orthosis with paravertebral/lumbosacral air chamber pads; (2) the same orthosis without air chamber pads; and (3) placebo body stocking. Measurements were performed with a 3D real-time ultrasound topometry system (Zebris (R) CMS 70). The posture correction was significantly more marked in the first group as compared to the second and third groups. The orthosis with air chamber pads causes a clinically meaningful trunk support in patients with osteoporotic posture changes. Since the device contains no rigid stabilizing elements, the change in posture was considered to be a result of muscle activation due to sensorimotor stimulation by the air chamber pads [27].

Another trial was conducted in 2012 evaluating the influence of Thämert Osteo-med spinal orthosis on gait and physical functioning in osteoporotic women with postmenopausal osteoporosis. At a 6-month followup, the study demonstrated that wearing a spinal orthosis reduced double support time associated with improvement on gait stability. Furthermore, there was also positive effect on pain-related restrictions of ADL. Beside reductions in pain and improvements in back extensor strength and correction of posture, the application of a spinal orthosis might induce advantages for gait stability and physical functioning in women with postmenopausal osteoporosis [33]. Pfeifer and his colleagues conducted a randomized study to evaluate the efficacy of two newly developed spinal orthoses in patients with osteoporotic vertebral fractures. Wearing the orthosis Spinomed in that study was associated with increase in back extensor and abdominal flexor strength, decrease in the angle of kyphosis and body sway, and also decrease in average pain. In addition, a better quality-of-life was achieved by pain reduction, decreased limitations of daily living, and improved the well-being [34].

In all above, mentioned studies, spinal orthosis had positive effects on postural balance which is in agreement with the results of our studies indicating improvement in FR and TUG test.

The only test which was not improved in WKO orthosis was unilateral stance test. One explanation can be relative small number of patients in intervention group compared to control group. The other explanation may be the fact that US only tests one aspect of balance-that is maintaining equilibrium on a reduced base of support. However, the greater task constraints and the need to cope with greatly increased postural sway may challenge a particular aspect of balance that is needed in saving a person during potential fall [25]. The US quantifies postural sway velocity with the patient standing on either the right or left foot on the floor. FR and TUG tests assess the aspects of balance that is different from US test. These tests altogether examine several different aspects of balance.

It may be speculated that WKO accompanied by home-based daily exercise program improved the aspects of balance which can be assessed by TUG and FR tests and not US test.

In conclusion, applying WKO together with back extensor strengthening exercises in women with osteoporosis leads to improvement in functional balance test which can be translated to decreased risk of fall-in real life in this population.

The limitations of our study were the relatively small number of cases included and short term follow ups evaluations. Absence of a control group receiving no intervention was another limitation of this study; however, due to ethical considerations we had to consider the least routine interventions including exercise and medications for all patients.

The potential bias which could arise by performing similar exercises in both groups was minimal due to the fact that these exercises needed more time (in comparison to our intervention duration) to cause significant clinical changes [18].

We encourage more randomized controlled clinical trials with larger sample size evaluating the effect of WKO on risk of falls in long term via applying clinical functional and paraclinical tests in the elderly with osteoporosis.

## Figures and Tables

**Table 1:** Demographic characteristics including age, height, weight, and body mass indices compared between two groups.

	Case group (*n* = 11) Mean ± SD	Control group (*n* = 20) Mean ± SD	*P* value
Age (years )	65.63	62.45	0.177
Height (cm)	150.45	152.25	0.47
Weight (Kg)	68.81	68.20	0.89
Body mass index (Kg/M^2^)	30.41	29.40	0.58

SD: standard deviation; case group (intervention group): orthosis and exercise; control group: exercise only.

**Table 2:** Functional scores compared between two groups before and after study.

	Case group	Control group	*P* value
TUG			
Before Tx	(*n* = 11) 9.28 ± 2.25	(*n* = 20) 9.20 ± 1.80	0.91
After Tx	(*n* = 9) 7.14 ± 1.10	(*n* = 19) 8.40 ± 1.03	0.00
*P* value	0.00	0.01	
FR			
Before Tx	(*n* = 11) 25.04 ± 2.69	(*n* = 20) 23.66 ± 3.56	0.91
After Tx	(*n* = 9) 31.64 ± 2.61	(*n* = 19) 26.43 ± 2.84	0.00
*P* value	0.00	0.00	
US			
Before Tx	(*n* = 11) 16.08 ± 8.20	(*n* = 20) 18.20 ± 10.01	0.55
After Tx	(*n* = 9) 18.18 ± 7.74	(*n* = 19) 18.81 ± 9.06	0.85
*P* value	0.30	0.00	

TUG: timed up and go test; FR: functional reach test; US: unilateral stance test; A: after trial, B: before trial.
